# The Effect of the Pore Entrance on Particle Motion in Slit Pores: Implications for Ultrathin Membranes

**DOI:** 10.3390/membranes7030042

**Published:** 2017-08-10

**Authors:** Armin Delavari, Ruth Baltus

**Affiliations:** Department of Chemical & Biomolecular Engineering, Clarkson University, Potsdam, NY 13699-5705, USA; delavaa@clarkson.edu

**Keywords:** membrane transport models, ultrathin membranes, ultrafiltration, microfiltration, pore entrance

## Abstract

Membrane rejection models generally neglect the effect of the pore entrance on intrapore particle transport. However, entrance effects are expected to be particularly important with ultrathin membranes, where membrane thickness is typically comparable to pore size. In this work, a 2D model was developed to simulate particle motion for spherical particles moving at small Re and infinite Pe from the reservoir outside the pore into a slit pore. Using a finite element method, particles were tracked as they accelerated across the pore entrance until they reached a steady velocity in the pore. The axial position in the pore where particle motion becomes steady is defined as the particle entrance length (PEL). PELs were found to be comparable to the fluid entrance length, larger than the pore size and larger than the thickness typical of many ultrathin membranes. Results also show that, in the absence of particle diffusion, hydrodynamic particle–membrane interactions at the pore mouth result in particle “funneling” in the pore, yielding cross-pore particle concentration profiles focused at the pore centerline. The implications of these phenomena on rejection from ultrathin membranes are examined.

## 1. Introduction

With the development of many new fabrication techniques, there has been an increasing interest in using ultrathin membranes in water purification, for biological, pharmaceutical and other separations as well as for sensing devices [1,2,3,4,5,6,7,8,9,10,11,12,13]. The thickness of these membranes is generally tens to several hundred nanometers, orders of magnitude smaller than the thickness of conventional membranes made using phase inversion and track-etch techniques. Ultrathin membranes allow for higher permeabilities with lower applied pressure compared to conventional membranes.

The degree of separation in porous membranes is typically characterized using the rejection coefficient, *σ_f_*, defined by [14]:

(1) $$\sigma_{f}=1-{\left[\frac{N}{C_{\infty }J_{v}}\right]}_{\Delta C_{\infty }=0}$$

where *N* is the particle flux, *J_v_* is the solvent velocity across the membrane and *C*_∞_ is the particle concentration in the feed. A general expression for rigid particles is developed by averaging the particle velocity over the pore cross-section:

(2) $$\sigma_{f}=1-\frac{\langle \frac{\vec{u}}{4\pi }\iint G\cdot \exp\left(-\frac{E}{k_{B}T}\right)d\hat{e}\rangle }{\langle \vec{u}\rangle }$$

where $\vec{u}$ is the fluid velocity in the absence of particles in the pore, $\hat{e}$ is the orientation of the particle in the pore, *G* is the hydrodynamic lag coefficient and *E* is the interaction energy between the particle and the pore wall. The brackets < > are defined as an average over the pore cross-sectional area.

Equation (2) is focused on particle transport within a pore and is based on the assumption that thermodynamic equilibrium is established at the pore mouth. This means that a Boltzmann expression can be used to give the time-averaged probability of finding a particle at a given position and orientation in the pore. When only steric interactions are considered, the probability of finding a particle at a sterically allowed location in the pore is 1 (*E* = *0*) and is 0 (*E* = ∞) for disallowed positions and orientations [14]. A Boltzmann distribution in the pore is valid when the particles have sufficient time to diffuse radially inside the pore and sample all allowed positions and orientations as they are carried through the pore. For small particles and long pores, this assumption is easily satisfied. However, its validity for ultrathin membranes is questionable.

Equation (2) is also based on the assumption that fluid travels at a steady velocity in the pore. At the pore entrance, fluid is funneled from a reservoir where it has a relatively low velocity and accelerates as it enters the pore, reaching a steady velocity after it travels a distance we define as the ‘fluid entrance length’ (FEL). Fluid entrance effects for flow into cylindrical tubes and channels has been an extensively studied problem where the distance required for flow to transition from plug flow (flat profile) to parabolic flow has been determined using both theoretical and experimental approaches. For flow into parallel plate channels, the FEL can be estimated using [15,16].

(3) FELh/2=1.25+0.088Reh

where *h* is the width of the channel and *Re_h_* is defined with the channel width as the characteristic length scale. Equation (3) shows that when *Re_h_* is small, the FEL is of order of the pore ½ width. When the pore length (i.e., membrane thickness) and pore size are of comparable magnitude (as is the case with ultrathin membranes [4,6,8,10,17]), Equation (3) predicts that the fluid profile may not be fully developed within the membrane, making Equation (2) of questionable validity for estimating particle rejection.

Previous studies of pore entrance effects have generally focused on particle motion in the reservoir outside the pore, examining the balance of forces controlling particle motion as it approaches a pore. The impact of different pore entrance shapes was studied by Bowen and Sharif [18] who used a finite element approach to relate the hydrodynamic and electrostatic forces on a spherical particle to the critical velocity of the system. Interactions of the particle with the pore walls at the entrance were studied for different pore mouth geometries; results showed that a rounded pore entrance has the highest critical velocity and is therefore the optimal choice.

Kao et al. [19] studied the impact of the pore entrance on trajectories of microscale particles by considering the influence of inertial and molecular forces for different Stokes number, showing that for low Stokes number, particle trajectories are affected largely by hydrodynamic forces. Other studies of pore entrance effects investigated particle–particle interactions in the vicinity of the pore entrance [20,21], the impact of electrostatic, Brownian and hydrodynamic forces on particle trajectories [22,23] and the effect of pore entrance size on the mass transfer of gas molecules [24,25].

As noted, previous studies have generally focused on particle transport within a pore or on the motion of a particle as it approaches a pore. In this study, we couple these to examine the impact of the pore entrance on intrapore particle transport. We report results from a model describing spherical particle motion from the reservoir outside the membrane into a slit pore. Solution of the coupled equations for fluid and particle motion yields the particle entrance length (PEL). Analogous to the FEL, the PEL is defined as the axial distance traversed by the particle within the pore before it achieves a steady velocity. The relationship between the PEL and the FEL, relative particle size, pore entrance geometry and *Re_h_* are examined and discussed in the context of ultrathin membranes where pore entrance effects are expected to be important.

## 2. Theoretical Model

### 2.1. Modeling Domain

**Figure 1:**
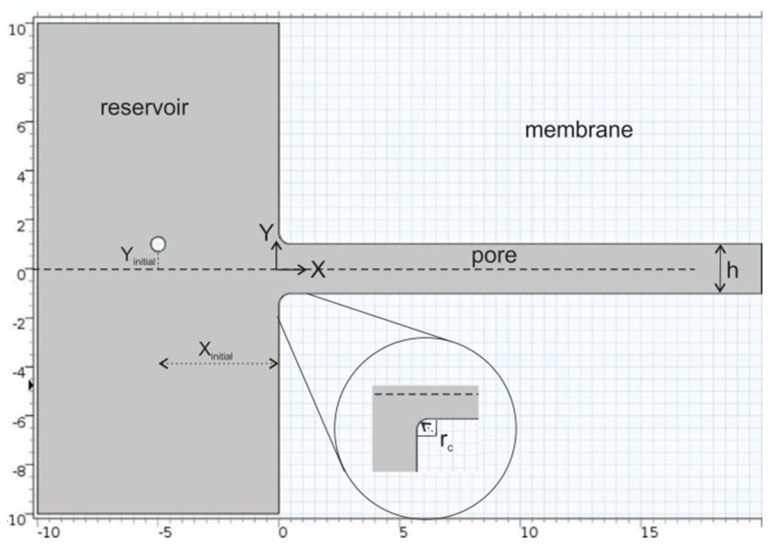
2D modeling domain: single-channel pore which is infinite in extent in the *Z* direction. The position variables *X* and *Y* are dimensionless, defined with respect to the pore ½ width, *h/2*. This image only shows a portion of the reservoir; the actual domain spanned from *Y* = −24 to *Y* = +24.

Particle motion from a reservoir into a slit pore was modeled in a 2D domain, with the system shown in Figure 1.

### 2.2. Immersed Boundary Method

The immersed boundary method was used to simulate particle motion from the reservoir into the pore [26]. In this approach, the particle was considered in a Lagrangian frame; an Eulerian perspective was used for the surrounding fluid. Using the Arbitrary Lagrangian–Eulerian concept, an appropriate mesh map was generated in the domain. To compute the forces imposed on the particle surface, a finite element method was employed to solve the Navier–Stokes equation coupled with Newton’s equation of motion throughout the 2D domain. In this approach, the particle boundaries consist of multiple nodes, with the number of nodes dependent on the mesh resolution. This approach enables one to appropriately include steric exclusion and the coupled motion of fluid and particle (i.e., the fluid impacts particle trajectory and particle motion impacts the fluid).

### 2.3. Model Equations

The particle trajectory was determined by solving Newton’s equation of motion in the absence of Brownian motion.

(4) $$m_{particle}\frac{d{\vec{u}}_{particle}}{d\;t}={\vec{F}}_{hydrodynamic}$$

where *m_particle_* is the particle mass and ${\vec{F}}_{hydrodynamic}$ is the hydrodynamic force on the particle surface. The Navier–Stokes and continuity equations were used to simulate fluid flow:

(5) $$\rho_{fluid}\left(\frac{\partial \vec{u}}{\partial t}+\vec{u}\cdot \vec{\nabla }\vec{u}\right)=\vec{\nabla }\cdot \left[-P\vec{I}+\mu \left(\nabla \vec{u}+{\left(\nabla \vec{u}\right)}^{T}\right)\right]$$

(6) $$\rho_{fluid}\nabla \cdot \vec{u}=0$$

The hydrodynamic force on the particle surface was determined by integration of the normal and shear stresses over the particle surface:

(7) $${\vec{F}}_{Hydrodynamic}=\iint \left[-P\vec{I}+\mu \left(\nabla \vec{u}+{\left(\nabla \vec{u}\right)}^{T}\right)\right]\cdot \vec{n}dS$$

where $\vec{n}$ is the normal vector on the particle surface.

The particle diameter, *d_particle_*, and the fluid velocity at the pore centerline, *u_o_*, were used as scaling parameters to non-dimensionalize these equations:

(8) u=u⇀uo ∇¯=dparticle∇ t¯=tuodparticle P¯=Pdparticleuoμm¯particle=mparticleuoμdparticle2 Redparticle=ρfluiduodparticleμ S¯=Sdparticle2

Note that the characteristic length in Redparticle is the particle diameter, different than Reh where the channel width is the characteristic length (Equation (3)). The dimensionless forms of equations of Equations (4)–(6) are

(9) $${\bar{m}}_{particle}\frac{du_{particle}}{d\bar{t}}=\frac{{\vec{F}}_{hydrodynamic}}{\mu d_{particle}u_{o}}$$

(10) Redparticle(∂u∂t¯+u⋅∇u)=∇⋅[−P¯I⇀+(∇u+(∇u)T)]

(11) $$\bar{\nabla }\cdot u=0$$

and the dimensionless hydrodynamic stress is:

(12) $$\frac{{\vec{F}}_{hydrodynamic}}{\mu d_{particle}u_{o}}=\iint \left[-\bar{P}\vec{I}+\left(\nabla u+{\left(\nabla u\right)}^{T}\right)\right]\cdot \vec{n}d\bar{S}$$

Particle diffusion in both cross-pore and axial directions is neglected in this analysis. Particle–particle interactions are also neglected. While the particle diameter was used as the length scale to non-dimensionalize the governing equations, results are presented with the pore ½ width as the length scale to non-dimensionalize positions in the pore and in the reservoir outside the pore.

For all calculations, a parabolic velocity profile was defined at the reservoir inlet (*X* = −10) and the pressure at the pore outlet was set to zero. While the reservoir in the image in Figure 1 spans from *Y* = −10 to *Y* = +10, the reservoir in our simulations spanned from *Y* = −24 to *Y* = +24. A no-slip condition was applied on the particle surface, on the pore wall and on the reservoir boundaries. With the reservoir width 24 times larger than the pore width, the reservoir boundaries have minimal effect on the flow into the pore. At time *t* = 0, a spherical particle was placed at position *X_initial_*, *Y_initial_* in the reservoir outside the pore.

### 2.4. Model Parameter Values

For most simulations, the average fluid velocity at the reservoir inlet (*X* = −10) was 4.08 × 10^−4^ m/s, which was based on a volumetric flow rate of 0.5 mL/min, typical of filtration measurements with track-etched membranes [27]. This leads to an average velocity in the pore of 0.0098 m/s and Redparticle is of *O*(10^−4^). Our results will show that the particle trajectories and the PEL are independent of velocity when the fluid velocity is <0.1 m/s. The pore length was set to 20 times the pore ½ width, which was found to be sufficient for the particle to reach a steady velocity in the pore. The radius of curvature of the pore mouth, *r_c_*, varied from 10 to 500% of the pore ½ width, with most calculations performed with *r_c_* = *h*/2. Calculations were limited to spherical particles, with dimensionless size *λ* = 0.1 to 0.9, where *λ* is defined as *d_particle_/ (h/2)*. Particle trajectories were determined for particles with specific gravity = 1.1, considering these to be representative of viruses and other bioparticles or macromolecules. Some simulations involved particles with specific gravity = 11, considering these to be representative of metallic nanoparticles. Simulations were repeated with different initial particle positions in the reservoir, *X_initial_* = −1 to −9 and *Y_initial_* = 0 to 5. The largest *Y_initial_* was chosen to be 5 because the pore-to-pore distance in track-etched membranes is estimated to be ~10 times the pore radius.

### 2.5. Solution of Model Equations

Equations (9)–(12) were solved simultaneously using COMSOL Multiphysics (COMSOL, Inc, Burlington, MA, USA), yielding particle position and velocity as the particle moves from the reservoir into the pore. Mesh refinement was performed to prevent distorted elements.

The particle accelerates as it moves from the reservoir into the pore and eventually reaches a steady velocity. The total force on the particle reaches its maximum when the particle passes the entrance of the pore (*X* = 0) and gradually declines as the particle attains its steady velocity. The particle entrance length (PEL) is defined as the axial particle position (*X*) where the particle has achieved a velocity 99.9% of its steady velocity.

The cross-sectional particle concentration profile at the PEL was determined by repeating simulations with 102 particles uniformly distributed in the reservoir.

## 3. Results and Discussion

### 3.1. Particle Trajectories

**Figure 2:**
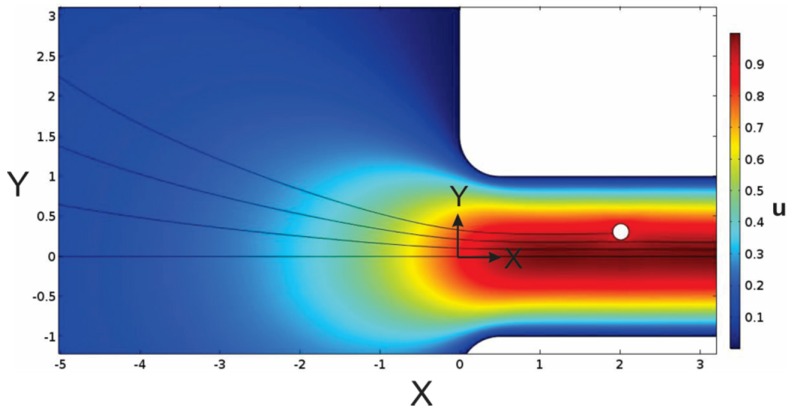
Velocity profile of fluid and particle trajectories for particles with *λ* = 0.1 and specific gravity = 1.1, initially placed in the reservoir *at X_initial_* = −5 and *Y_initial_* = 0, 0.6, 1.4 and 2.2. For these simulations, *r_c_* = *h*/2.

The trajectories of four particles are illustrated in Figure 2 where the particles were initially placed at *X_initial_* = −5 and *Y_initial_* = 0, 0.6, 1.4 and 2.2. Note that the computational domain is bounded at *X* = −10 (see Figure 1), with a parabolic velocity profile prescribed at this boundary. This surface has width = 48 dimensionless length units, whereas the pore width is 2 dimensionless length units. The reservoir is sufficiently large such that the velocity profile is approximately flat until it is influenced by the pore entrance (at *X* ≈ −2).

### 3.2. Particle Velocities

**Figure 3:**
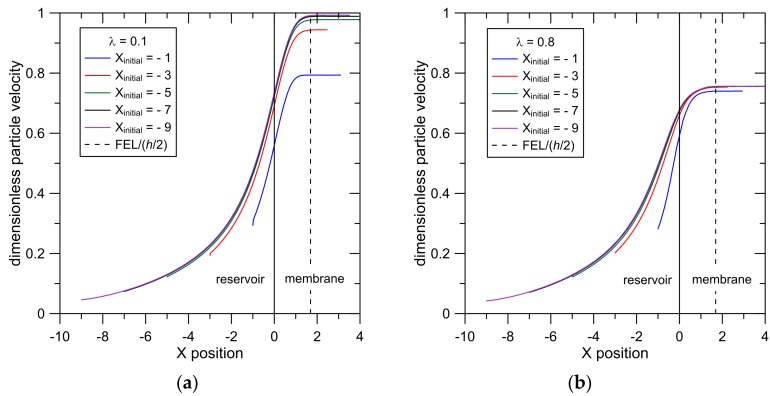
Particle velocity as a function of *X* for particles moving from the reservoir into the pore for (**a**) *λ* = 0.1 and (**b**) *λ* = 0.8; particle specific gravity = 1.1, *Y_initial_* = 1 and *X_initial_* = −1, −3, −5, −7 −9.

The effect of the initial particle placement in the reservoir on particle velocity is presented in Figure 3a for particles with *λ* = 0.1 and in Figure 3b for particles with *λ* = 0.8. Here, particles were initially placed in the reservoir at *Y_initial_* = 1 and *X_initial_* = −1, −3, −5, −7 and −9. These results illustrate the acceleration experienced by the particles as they approach and enter the pore. Results show that particle trajectories are indistinguishable when −9 < *X_initial_* < −5 because particles initially placed more than five pore ½ widths from the membrane surface get carried into the pore along the same streamlines. Results also show that the steady intrapore particle velocity increases as |*X_initial_*| increases because particles initially placed farther from the membrane surface are carried towards the pore centerline.

The larger particles (*λ* = 0.8, Figure 3b) show a smaller and tighter range of intrapore particle velocities when compared to the smaller particles (*λ* = 0.1, Figure 3a). This results from larger hydrodynamic resistances experienced by the larger particles and from steric constraints which restrict the larger particles to centerline positions (−0.6 < *Y* < 0.6).

### 3.3. Fluid Entrance Length

Simulations were also performed with a particle-free system and the fluid velocity profile in the pore was examined. The FEL was defined as the axial (*X*) position in the pore where the axial velocity was 99.9% of its fully developed value (where velocity is independent of axial position). Results for FEL/(*h*/2) as a function of *Y* are shown in Figure S1 in the Supplementary Information. These results show that FEL/(*h*/2) values are ~constant across the pore (FEL/(*h*/2) = 1.65 to 1.75), except at *Y* = 0.3–0.4 (minimum FEL/(*h*/2) = 1.26 at *Y* = 0.4). To transition from a relatively flat velocity profile at *X* = 0 to a steady parabolic profile at *X* = PEL, the fluid velocity at the centerline increases and the fluid velocity near the pore wall decreases. Between these two regions, the fluid velocity does not change significantly; hence the minimum FEL at this point.

To be consistent with previous reports of entrance lengths [15,16], we define FEL based on the velocity at the pore centerline, where FEL/(*h*/2) = 1.69. This is larger than the value predicted from Equation (3) (FEL/(*h*/2) = 1.25). As in other previous studies of entrance lengths, the FEL reported in Equation (3) was based on a centerline velocity equal to 99% of the value at fully developed flow, whereas our results were determined with a tighter criterion (99.9%). When the criterion is set to 99%, our simulations yield a centerline FEL/(*h*/2) = 1.28, in close agreement with Equation (3). In addition, Equation (3) was derived by considering boundary layer flow near the channel wall when flow transitions from a flat profile to a parabolic flow [15], a situation which is similar, but not the same as the problem considered here. In this paper, we use the centerline value FEL/(*h*/2) = 1.69 and this value is included in Figure 3.

### 3.4. Particle Entrance Lengths

**Figure 4:**
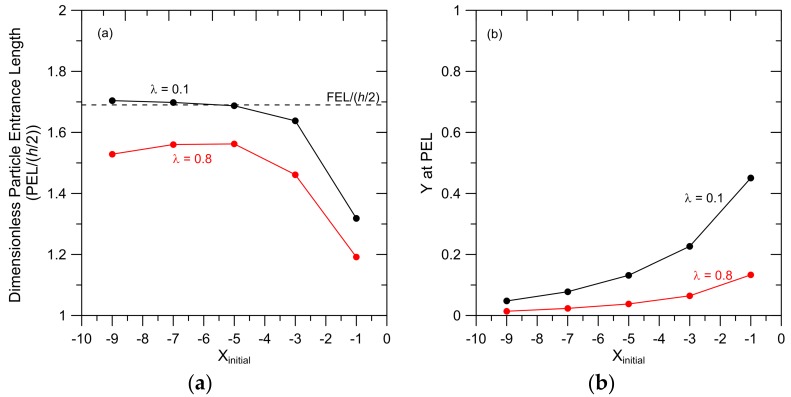
(**a**) Particle entrance length (PEL) as a function of *X_initial_* and (**b**) *Y* at PEL as a function of *X_initial_* for particles with *λ* = 0.1 and *λ* = 0.8. Particles were initially placed at *Y_initial_* = 1 and particle specific gravity = 1.1. The radius of curvature of the pore entrance, *r_c_*/(*h*/2) = 1.

The particle entrance length (PEL) and the cross-pore particle position (*Y*) at the PEL were determined by examining the results presented in Figure 3; results are presented in Figure 4. For both *λ* = 0.1 and *λ* = 0.8, the PEL is generally independent of *X_initial_* when *X_initial_* = −5, −7 and −9 (Figure 4a); these particles also reach approximately the same cross-pore position in the pore (Figure 4b). These results arise because particles that are initially placed 5 or more pore ½ widths from the membrane surface follow approximately the same trajectories into the pore.

Particles initially placed closer to the membrane surface (*X_initial_* = −1 and −3) have smaller PEL and are carried closer to the pore wall when compared to those initially placed farther from the membrane. Particles that are carried closer to the pore wall achieve a smaller steady velocity (Figure 3). With a smaller change in particle velocity between *X* = 0 and *X* = PEL, these particles achieve a steady velocity in less time (and therefore in a shorter distance) when compared to particles initially placed farther from the membrane face.

The results in Figure 4a show that PEL for the smaller particles is greater than the PEL for the larger particles. This can again be explained by considering the smaller change in velocity experienced by the larger particle when it travels from the pore entrance to the PEL. Particles with *λ* = 0.1 and placed initially at *X_initial_* = −5 undergo a 35% increase in velocity from *X* = 0 to *X* = PEL whereas the velocity of the larger particles increases by only 12% from *X* = 0 to *X* = PEL.

The difference between the PEL and FEL for *λ* = 0.1 particles that are placed at *X_initial_* = −1 can be explained by recognizing that these particles achieve their steady velocity at *Y* = 0.45 (Figure 4b). As shown in Figure S1 (Supplementary Information), this is the pore region where the FEL is considerably smaller than the centerline value (FEL/(*h*/2) = 1.26 at *Y* = 0.4). The difference between the FEL and the PEL is relatively large because the FEL value presented in Figure 4a is based on the centerline velocity. The relatively small value for the PEL for the *λ* = 0.8 particles that are placed at *X_initial_* = −1 arises from the fact that the steady velocity reached by these particles is relatively small because they reach that steady velocity at *Y* = 0.13, closer to the pore wall than for the same-sized particles initially placed farther from the membrane surface. Again, a smaller steady velocity means less time and a shorter distance is required for the particle to reach the PEL.

The results presented in Figure 4 demonstrate that results for particles that approach the membrane from five or more ½ pore widths from the surface are essentially the same. Therefore, in subsequent simulations, particles were placed at *X_initial_* = −5. This should be a reasonable representation of particle trajectories to a membrane pore from a reservoir.

**Figure 5:**
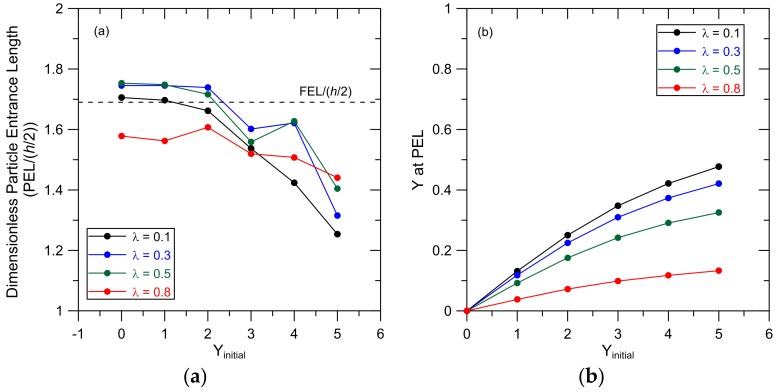
(**a**) PEL as a function of *Y_initial_* and (**b**) *Y* at PEL as a function of *Y_initial_* for particles with *λ* = 0.1, 0.3, 0.5 and 0.8. Particles were initially placed at *X_initial_* = −5 and particle specific gravity = 1.1. The pore centerline is at *Y* = 0 (Figure 1). The radius of curvature of the pore entrance, *r_c_*/(*h*/2) = 1.

The dependence of the PEL on *Y_initial_* was examined for particles with relative size *λ* = 0.1, 0.3, 0.5 and 0.8 and *X_initial_* = −5; results are shown in Figure 5a. The cross-sectional particle position (*Y*) at the PEL as a function of *Y_initial_* from these simulations is shown in Figure 5b. Particles that are initially placed at *Y_initial_* = 0 maintain this cross-pore position as they are carried into and through the pore because particle diffusion is neglected and there is a symmetric flow field around the particles in the *Y* direction. In general, the smaller particles (*λ* = 0.1, 0.3 and 0.5) have similar PEL values, which are generally independent of *Y_initial_* for particles initially placed at *Y_initial_* = 0, 1 and 2. The PEL for these particles decreases and the cross-pore position at the PEL increases (Figure 5b) when particles are initially placed in the reservoir farther from the pore centerline (*Y_initial_* = 3, 4 and 5). These trends can again be explained by recognizing that these particles are carried into the pore at *Y* positions close to the pore wall (larger *Y* at the PEL) and therefore achieve smaller particle velocities compared to particles initially placed at smaller *Y_initial_*. The relationship between PEL, *Y_initial_* and *λ* appears to be complex when *Y_initial_* = 3, 4 and 5, particularly for the smaller particles (*λ* = 0.1, 0.3 and 0.5). These results arise because these particles reach their PEL between *Y* = 0.25 and 0.47. This is the area in the pore where the FEL shows a minimum (Figure S1 in Supplementary Information). Therefore, the confluence of different steric constraints and hydrodynamic interactions influencing the different-sized particles and the fluid response to the pore entrance (FEL(*Y*)) leads to complex relationships between PEL and particle size and *Y_initial_*. The PELs for the larger particles (*λ* = 0.8) are less sensitive to *Y_initial_* than the smaller particles. Since steric constraints limit available positions in the pore for these particles, *Y* at the PEL is also less sensitive to *Y_initial_*, leading to particle velocities, and therefore PELs, that vary little with *Y_initial_*.

A general conclusion that can be drawn from the results presented in Figure 4 and Figure 5 is that as *Y* at the PEL increases, the PEL decreases. This trend can be attributed to the smaller velocities achieved by particles when they are carried towards the pore wall.

Finally, the results in Figure 4b and Figure 5b show that hydrodynamic forces leave a significant portion of the pore free of particles. For example, equilibrium steric restrictions allow particles with *λ* = 0.1 to sample cross-sectional positions from *Y* = −0.95 to *Y* = 0.95, but our results show that, without diffusion, these particles will be found within −0.5 < *Y* < 0.5. This ‘funneling’ phenomenon is addressed in further detail in the subsequent discussion of particle concentration profiles.

### 3.5. Particle Concentration Profiles

**Figure 6:**
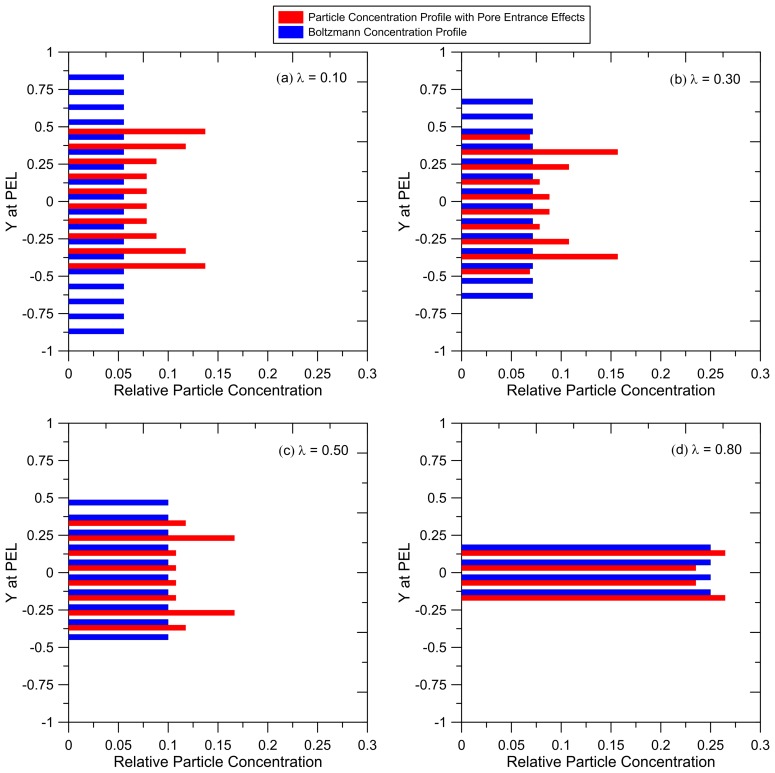
Relative particle concentration profile at the PEL predicted from our pore entrance model compared to a Boltzmann probability for different particle sizes: (**a**) *λ* = 0.10; (**b**) *λ* = 0.30; (**c**) *λ* = 0.50; (**d**) *λ* = 0.80. Simulations were repeated with 102 particles for each particle size, initially placed at *X_initial_* = −5 and evenly distributed between −5 < *Y_initial_* < +5. The radius of curvature of the pore entrance, *r_c_*/(*h*/2) = 1.

Simulations were repeated with 102 particles that were initially placed at *X_initial_ =* −5 and were uniformly distributed in the reservoir between *Y_initial_* = −5 and *Y_initial_ =* 5. The trajectories of these particles were tracked as they reached a steady velocity in the pore channel. These simulations were repeated with collections of particles with different size (*λ* = 0.1, 0.3, 0.5 and 0.8). The number of particles in each downstream cross-sectional pore segment (at the PEL) were counted, yielding particle concentration profiles shown in Figure 6. These concentration profiles are compared to the uniform profile predicted for a Boltzmann distribution. These results show that hydrodynamic particle–pore entrance interactions ‘funnel’ the particles towards the pore center. The difference between the profiles predicted from our pore entrance model and the uniform Boltzmann profile is most pronounced for the smallest particles (*λ* = 0.1). Equilibrium steric constraints restrict these particles to −0.95 < *Y* < 0.95, but results show that the hydrodynamic forces place these particles only within the window −0.5 < *Y* < 0.5. The particles are most concentrated at the outer edge of this window because all particles initially placed at larger *Y* in the reservoir are carried into these regions in the pore (as shown in Figure 5b). For larger particles, the difference between the two profiles is reduced, with only small differences for particles with *λ* = 0.8, because these particles are restricted by steric constraints to a narrow window near the pore centerline.

The results in Figure 6 show that, in the absence of particle diffusion, particles at the PEL are concentrated closer to the pore centerline than is predicted from a Boltzmann probability. For thick membranes and small particles, sufficient residence time in the pore is expected to enable particles to diffuse across the pore and a Boltzmann profile will develop within a relatively short distance, enabling one to use Equation (2) to predict rejections. However, that may not be the case for ultrathin membranes with thickness that is at most ~ten times the pore size.

In a membrane with thickness *L* and pore size *h*, the convective transport time is *L/u* and cross-pore diffusive transport time is *h*^2^*/D*, where *D* is the particle diffusivity which can be estimated using the Stokes–Einstein equation (*D* = *k_B_T*/(3 π µ d*_particle_*)). Equating the convective to diffusive transport times, one can estimate the pore length (membrane thickness) required for diffusive forces to counter the particle “funneling” caused by hydrodynamic interactions at the pore entrance. For a 20 nm diameter particle entering a 200 nm pore (*λ* = 0.1), the convective and diffusive (cross-pore) transport times are estimated to be equal when the pore length is ~16 µm. For a symmetric conventional porous UF membrane, this distance is perhaps 10% of the entire pore length. Therefore, traditional particle rejection models which are based on a Boltzmann concentration profile are expected to be valid. However, ultrathin membranes generally have thicknesses that are less than several hundred nm; asymmetric membranes have selective layers which are ~1 µm thick. For these systems, the results in Figure 6 and our calculations of particle transit and cross-pore diffusion times indicate that particle residence time in the pore is likely to be less than the time required for a Boltzmann concentration profile to develop. The ‘non-equilibrium’ concentration profiles shown in Figure 6 should be considered when developing particle rejection models for these systems.

### 3.6. Effect of the Radius of Curvature of the Pore Entrance

**Figure 7:**
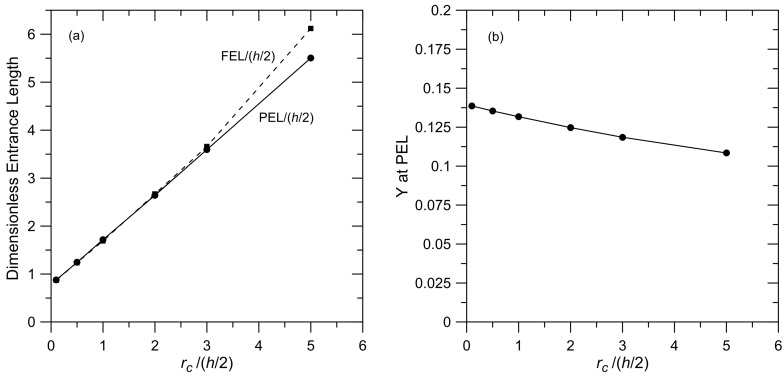
Effect of the radius of curvature at the pore mouth on (**a**) PEL and fluid entrance length (FEL) and (**b**) *Y* at the PEL. Results are shown for a system with *λ* = 0.1, particle specific gravity = 1.1, *Y_initial_* = 1 and *X_initial_* = −5.

Simulations were performed for membranes with different radius of curvature at the pore mouth for particles with *λ* = 0.1 that were initially placed in the reservoir at *Y_initial_* = 1 and *X_initial_* = −5. The relationship between PEL and FEL and the radius of curvature are shown in Figure 7a. These results show a strong correlation between the PEL and the FEL and the radius of curvature, with a linear relationship for the PEL and a nearly linear relationship for the FEL. The estimation for FEL in Equation (3) does not include the radius of curvature of the channel entrance because, as noted earlier, the analysis leading to this expression considers the transition from a flat velocity profile to a steady parabolic profile within a channel; it does not consider flow into the channel.

When *r_c_*/(*h*/2) < 3, there is close agreement between the PEL and the FEL, with both increasing by a factor of ~4 when *r_c_*/(*h*/2) increases from 0.1 (essentially a square corner) to 3. For larger (and perhaps unrealistic) *r_c_*, FEL > PEL. These results illustrate the importance of the geometry of the membrane face on effective entrance lengths. This geometry will be influenced by the process used to fabricate the membrane.

The cross-sectional position of the particle at the PEL for different *r_c_* is presented in Figure 7b. These results show a generally weak dependence on the final particle position and *r_c_*. We expect a stronger dependence for larger particles where steric restrictions will be more important. In our discussion of the results in Figure 4 and Figure 5, we noted the relationship between PEL and *Y* at PEL, with the PEL decreasing as particles get carried closer to the pore wall. The results in Figure 7 indicate that the relationship between PEL and *Y* at the PEL is perhaps more complicated. These results show a large increase in PEL when *r_c_*/(*h*/2) increases from 0.1 to 5 with only a small change in the particle position at the PEL.

### 3.7. Effect of Re_h_

**Figure 8:**
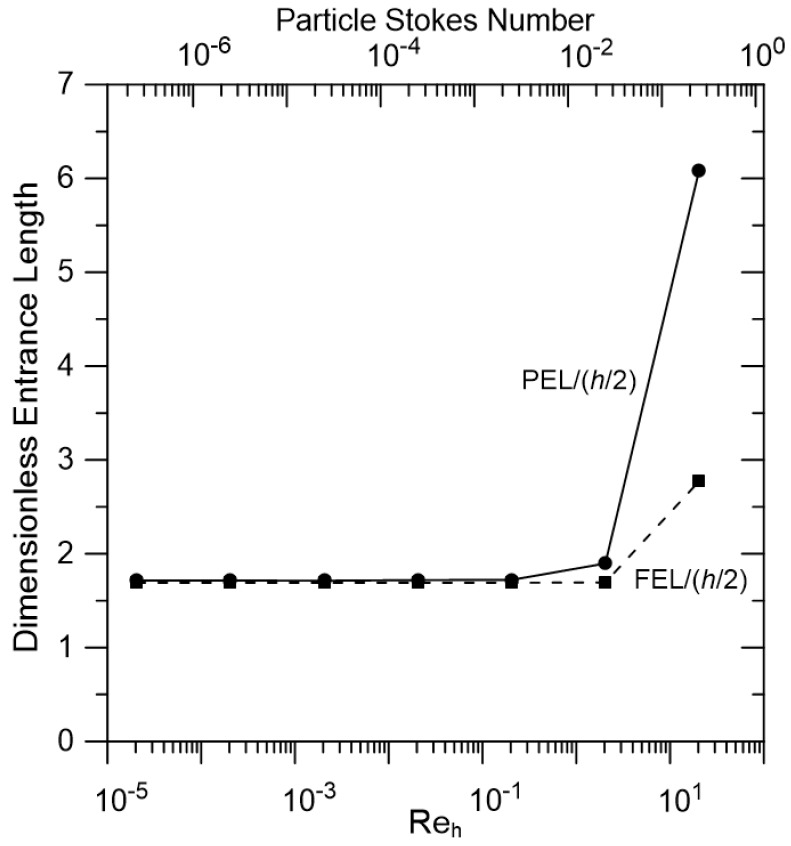
Effect of Reh on PEL and FEL. Results are presented for a system with *λ* = 0.1, particle specific gravity = 1.1, *Y_initial_* = 1, *X_initial_* = −5 and *r_c_*/(*h*/*2*) = 1.

The results presented in Figure 3, Figure 4, Figure 5, Figure 6 and Figure 7 were obtained for a specific reservoir velocity (4.08 × 10^−−4^ m/s, *Re_h_ =* 2 × 10^−5^). Simulations were also performed with higher fluid velocities; PEL and FEL versus *Re_h_* are shown in Figure 8. The particle Stokes number ((*ρ_particle_/ρ_fluid_*) (*d_particle_/h*) Redparticle) is also included in this figure. These results show that, for *Re_h_* < 2 (Stokes number < 0.03), the PEL and the FEL are both independent of *Re_h_*, with close agreement between the PEL and the FEL. Both the PEL and the FEL increase for larger *Re_h_*, with a much larger increase for the PEL compared to the FEL because of particle inertia at these conditions. Note that this increase in PEL occurs when the Stokes number is less than 1. While UF and MF separations are typically performed at low *Re_h_* conditions (*Re_h_* << 1), where inertial effects should not be important, the fact that PEL/FEL increases from ~1 at low *Re_h_* to ~2 when *Re_h_* > 10 is an interesting observation. The higher *Re_h_* results may also be important in gas filtration processes where viscous effects are generally not significant.

### 3.8. Effect of Particle Specific Gravity

**Figure 9:**
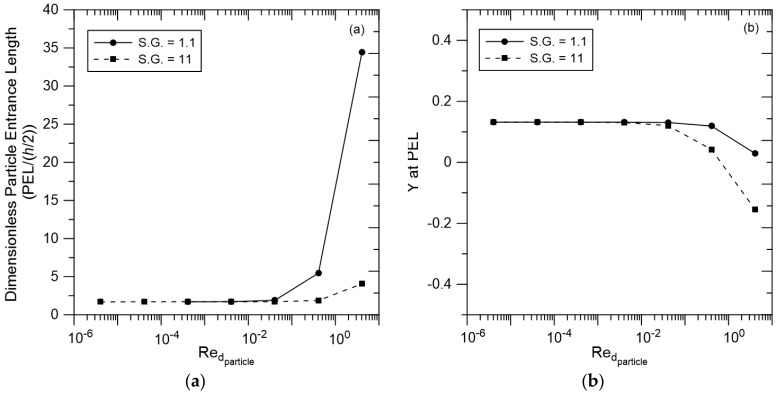
(**a**) PEL as a function of Redparticle for particles with specific gravity = 1.1 and 11; (**b**) *Y* at PEL as a function of Redparticle for these same particles. Results are presented for a system with *λ* = 0.1, *Y_initial_* = 1, *X_initial_* = −5 and *r_c_*/(*h*/*2*) = 1.

The results presented in Figure 3, Figure 4, Figure 5, Figure 6, Figure 7 and Figure 8 were obtained for particles with specific gravity = 1.1, characteristic of viruses and other bioparticles or macromolecules. Results for these particles are compared to results from simulations performed with particles with specific gravity = 11 in Figure 9, with PEL versus Redparticle shown in Figure 9a and *Y* at PEL versus Redparticle shown in Figure 9b. At Redparticle < 0.04, results for the two particles are essentially the same because transport at these conditions is governed by viscous forces and inertia is insignificant. These results illustrate that one can predict behavior for bioparticles (S.G. = 1.1) from measurements with metallic nanoparticles (S.G. = 11) or vice versa. The particle systems differ only when Redparticle > 0.1, conditions that are generally not experienced in membrane filtration with liquids, but may be important in gas separations.

Similarly, the results in Figure 9b show no difference between the steady cross-pore positions achieved by the two different particles when Redparticle is small. At higher Redparticle, inertia is important, yielding differences in *Y* at the PEL for the two particles. An interesting observation can be made for the results for the denser particle at Redparticle = 4. Here, the particle follows a trajectory that carries it to *Y* = −0.16; inertia has carried the particle across the pore centerline.

### 3.9. Pore Geometry

Our model neglects the impact of neighboring pores on particle motion into a pore, an assumption that may be questionable for high porosity membranes. To model higher porosity membranes, initial particle placement in the reservoir would be limited to smaller *Y_initial_* values where PEL is larger and particles will be carried close to the pore centerline (Figure 3 and Figure 5). Here, one would expect more pronounced particle ‘funneling’ than demonstrated with the current results. Therefore, the impact of the pore entrance is expected to be even more important for higher porosity membranes.

### 3.10. Particle Concentration/Feed Mixtures

The results presented here were obtained with a model in which particle–particle interactions were neglected, i.e., low feed concentrations are assumed. To simulate systems with higher particle concentrations requires that one add particle–particle interactions to the particle–membrane interactions that are included in the current model. This would add considerable complexity, and the concomitant computational time, to the simulations.

The results in Figure 4a show that the PEL increases as particle size decreases. Therefore, in order to put an upper bound on the PEL for a low concentration mixture containing particles of various sizes, one should consider the smallest particles in the mixture.

### 3.11. Implications for Rejection Models

As noted earlier, the general expression for membrane rejection (Equation (2)) is based on the assumptions that equilibrium is established at the pore entrance and that particle and fluid velocities are independent of axial position in the pore. The results reported here put into question the validity of these assumptions for ultrathin membranes.

The Boltzmann expression in Equation (2) involves potential particle–pore wall interaction energies. To correct the Boltzmann term for the non-steady particle and fluid velocities in the pore entrance region, particle kinetic energies can be added to the potential energy. We are currently developing a rejection model that incorporates axially dependent fluid and particle velocities and the non-uniform cross-pore concentration profiles that were determined in this study. Results will be forthcoming.

## 4. Conclusions

A 2D particle transport model was developed to examine spherical particle motion from a reservoir into a single-slit pore. Results show that particles accelerate as they travel into the pore and achieve a steady velocity a distance into the pore we define as the particle entrance length, PEL. The effects of relative particle size, pore entrance geometry and initial particle placement in the reservoir on the PEL were examined. The centerline fluid entrance length, FEL, was also determined for a particle-free system, with the FEL found to be 69% larger than the pore width when the radius of curvature of the pore mouth was equal to the pore ½ width. This value is larger than values reported in previous studies of fluid entrance lengths because we have placed a tighter criterion (99.9%) on agreement between centerline fluid velocity and the velocity with fully developed flow. The different system considered in this study (flow from a reservoir into a channel) compared to previous studies may also play a role in this difference.

Results show that, in general, the PEL is comparable to the FEL, with the PEL decreasing when particles are carried to intrapore positions closer to the pore wall. The relationship between PEL, particle size and initial particle placement in reservoir is complex because of the confluence of steric constraints, hydrodynamic particle–membrane resistances and the response of the fluid as it enters the pore. The PEL was found to depend strongly on the radius of curvature of the pore entrance, with the PEL increasing by a factor of ~6 when the radius of curvature increased from 10 to 500% of the pore ½ width.

In the absence of particle diffusion, hydrodynamic interactions between the particles and the pore entrance lead to ‘funneling’ of the particles towards the pore centerline, yielding particle cross-pore concentration profiles that can differ significantly from profiles predicted from a Boltzmann probability.

Ultrathin membranes can have thickness comparable to the pore size; therefore, pore entrance effects are expected to be particularly important for these systems. Results from this study indicate that particles may exit pores in these membranes before they achieve a steady velocity and before Boltzmann concentration profiles can develop by cross-pore diffusion. Therefore, conventional models may not accurately describe particle transport and rejections for these systems. We are currently developing an alternative approach to model such systems.

## Supplementary Materials

The following are available online at http://www.mdpi.com/2077-0375/7/3/41/s1, Figure S1: Dimensionless FEL as a function of cross-pore position (Y).

## References

[1] Striemer C.C., Gaborski T.R., Mcgrath J.L., Fauchet P.M. (2007). Charge-and size-based separation of macromolecules using ultrathin silicon membranes. Nature.

[2] Akbari A., Sheath P., Martin S.T., Shinde D.B., Shaibani M., Banerjee P.C., Tkacz R., Bhattacharyya D., Majumder M. (2016). Large-area graphene-based nanofiltration membranes by shear alignment of discotic nematic liquid crystals of graphene oxide. Nat. Commun..

[3] Ling S., Jin K., Kaplan D.L., Buehler M.J. (2016). Ultrathin free-standing bombyx mori silk nanofibril membranes. Nano Lett..

[4] Lee Y.M., Jung B., Kim Y.H., Park A.R., Han S., Choe W.S., Yoo P.J. (2014). Nanomesh-structured ultrathin membranes harnessing the unidirectional alignment of viruses on a graphene-oxide film. Adv. Mater..

[5] Zhu Y., Gao S., Hu L., Jin J. (2016). Thermoresponsive Ultrathin Membranes with Precisely Tuned Nanopores for High-Flux Separation. ACS Appl. Mater. Interfaces.

[6] Popa A.-M., Niedermann P., Heinzelmann H., Hubbell J.A., Pugin R. (2009). Fabrication of nanopore arrays and ultrathin silicon nitride membranes by block-copolymer-assisted lithography. Adv. Mater..

[7] Liu G., Jiang Z., Cheng X., Chen C., Yang H., Wu H., Pan F., Zhang P., Cao X. (2016). Elevating the selectivity of layer-by-layer membranes by in situ bioinspired mineralization. J. Memb. Sci..

[8] Fang D.Z., Striemer C.C., Gaborski T.R., McGrath J.L., Fauchet P.M. (2010). Methods for Controlling Pore Properties of Ultra-thin Nanocrystalline Silicon Membranes. J. Phys. Condens. Matter.

[9] Feinberg B.J., Hsiao J.C., Park J., Zydney A.L., Fissell W.H., Roy S. (2017). Silicon nanoporous membranes as a rigorous platform for validation of biomolecular transport models. J. Memb. Sci..

[10] Han Y., Xu Z., Gao C. (2013). Ultrathin Graphene Nanofiltration Membrane for Water Purification. Adv. Funct. Mater..

[11] Mireles M., Gaborski T.R. (2017). Fabrication techniques enabling ultrathin nanostructured membranes for separations. Electrophoresis.

[12] Wong H.C., Zhang Y., Viasnoff V., Low H.Y. (2017). Predictive Design, Etch-Free Fabrication of Through-Hole Membrane with Ordered Pores and Hierarchical Layer Structure. Adv. Mater. Technol..

[13] Warkiani M.E., Bhagat A.A.S., Khoo B.L., Han J., Lim C.T., Gong H.Q., Fane A.G. (2013). Isoporous micro/nanoengineered membranes. ACS Nano.

[14] Anderson J.L. (1981). Configurational effect on the reflection coefficient for rigid solutes in capillary pores. J. Theor. Biol..

[15] Deen W.M. (1998). Analysis of Transport Phenomena.

[16] Atkinson B., Brocklebank M.P., Card C.C.H., Smith J.M. (1969). Low Reynolds Number Developing Flows. AIChE J..

[17] Burgin T., Johnson D., Chung H., Clark A., McGrath J. (2015). Analytical and finite element modeling of nanomembranes for miniaturized, continuous hemodialysis. Membranes (Basel).

[18] Bowen W.R., Sharif A.O. (2002). Prediction of optimum membrane design: Pore entrance shape and surface potential. Colloids Surf. A Physicochem. Eng. Asp..

[19] Kao J.-N., Wang Y., Pfeffer R., Weinbaum S. (1988). A theoretical model for nuclepore filters including hydrodynamic and molecular wall interaction effects. J. Colloid Interface Sci..

[20] Agbangla G.C., Bacchin P., Climent E. (2014). Collective dynamics of flowing colloids during pore clogging. Soft Matter.

[21] Ramachandran V., Venkatesan R., Tryggvason G., Scott F.H. (2000). Low Reynolds Number Interactions between Colloidal Particles near the Entrance to a Cylindrical Pore. J. Colloid Interface Sci..

[22] Kim M., Zydney A.L. (2004). Effect of electrostatic, hydrodynamic, and Brownian forces on particle trajectories and sieving in normal flow filtration. J. Colloid Interface Sci..

[23] Bowen R.W., Filippov A.N., Sharif A.O., Starov V.M. (1999). A model of the interaction between a charged particle and a pore in a charged membrane surface. Adv. Colloid Interface Sci..

[24] Ford D.M., Glandt E.D. (1995). Steric hindrance at the entrances to small pores. J. Memb. Sci..

[25] Lim Y.-I., Bhatia S.K. (2011). Simulation of methane permeability in carbon slit pores. J. Memb. Sci..

[26] Peskin C.S. (2002). The immersed boundary method. Acta Numer..

[27] Agasanapura B., Baltus R.E., Tanneru C., Chellam S. (2013). Membrane rejection of nonspherical particles: Modeling and experiment. AIChE J..

