# Supplementary material for: The Effect of the Pore Entrance on Particle Motion in Slit Pores: Implications for Ultrathin Membranes
Source: Membranes (Basel). 2017 Aug 10;7(3):42. doi: 10.3390/membranes7030042 (PMC5618127; doi:10.3390/membranes7030042)
Supplement: Supplementary file 1 [file membranes-07-00042-s001.pdf]

Supplementary Information

The Effect of the Pore Entrance on Particle Motion in Slit Pores: Implications for Ultrathin Membranes

Armin Delavari and Ruth Baltus

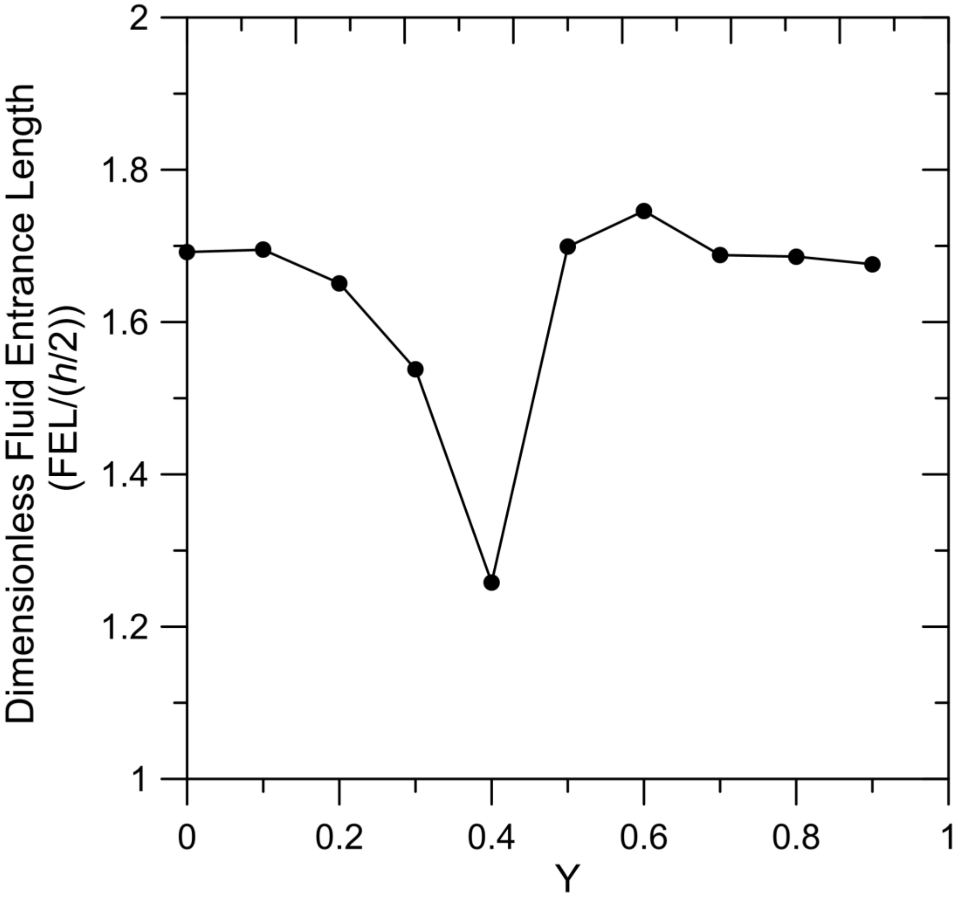

Figure S1 Dimensionless FEL as a function of cross-pore position (*Y*)
